# The utility of blood pressure variability as a biomarker

**DOI:** 10.1002/alz70856_098059

**Published:** 2025-12-24

**Authors:** Daniel A. Nation

**Affiliations:** ^1^ Leonard Davis School of Gerontology, University of Southern California, Los Angeles, CA, USA

## Abstract

Age‐related changes in the ANS contribute to increased blood pressure and hypertension, but may also contribute to the increase the variability in blood pressure from visit‐to‐visit, beat‐to‐beat, or even within a cardiac cycle. These fluctuations in blood pressure over various timescales have each been linked to increased risk for Alzheimer's disease and dementia, independent of average blood pressure, hypertension, or its treatment. Several questions remain as to the role and potential clinical utility of blood pressure variability. Does preclinical Alzheimer's disease lead to increased blood pressure variability through an ANS‐related mechanism? Does increased blood pressure variability causally contribute to Alzheimer's disease? Can blood pressure variability serve as a biomarker or modifiable treatment target in Alzheimer's disease?

